# A two-session psychological intervention for siblings of pediatric cancer patients: a randomized controlled pilot trial

**DOI:** 10.1186/1753-2000-6-3

**Published:** 2012-01-11

**Authors:** Alice Prchal, Anna Graf, Eva Bergstraesser, Markus A Landolt

**Affiliations:** 1 Department of Psychosomatics and Psychiatry, University Children's Hospital Zurich, Zurich, Switzerland; 2 Children's Research Center, University Children's Hospital Zurich, Zurich, Switzerland; 3 Department of Pediatric Oncology, University Children's Hospital Zurich, Zurich Switzerland

**Keywords:** Siblings, childhood cancer, oncology, intervention, psychosocial adjustment

## Abstract

**Background:**

Since siblings of pediatric cancer patients are at risk for emotional, behavioral, and social problems, there is considerable interest in development of early psychological interventions. This paper aimed at evaluating the effectiveness of a two-session psychological intervention for siblings of newly diagnosed pediatric cancer patients.

**Methods:**

Thirty siblings age 6-17 years were randomly assigned to an intervention group or an active control group with standard psychosocial care. The manualized intervention provided to siblings in the first 2 months after the cancer diagnosis of the ill child included medical information, promotion of coping skills, and a psychoeducational booklet for parents. At 4 to 6 weeks, 4 months, and 7 months after the diagnosis, all siblings and their parents completed measures (from standardized instruments) of social support, quality of life, medical knowledge, posttraumatic stress symptoms, and anxiety.

**Results:**

At follow-up siblings in the intervention group showed better psychological well-being, had better medical knowledge, and reported receiving social support from more people. However, the intervention had no effects on posttraumatic stress symptoms and anxiety.

**Conclusions:**

The results of this pilot trial suggest that a two-session sibling intervention can improve siblings' adjustment, particularly psychological well-being, in the early stage after a cancer diagnosis.

**Trial Registration:**

ClinicalTrials.gov NCT00296907

## Background

Having a brother or sister newly diagnosed with cancer is a distressing and challenging situation. The cancer diagnosis in the family has emotional, behavioral and social consequences for siblings [1]. Siblings are confronted with changed daily routines in the family and decreased physical and emotional availability of their parents [1,2]. They are worried about the illness and have to observe their brother or sister have emotional and physical pain. These experiences may lead to intrusive and conflicting emotions such as fear, loneliness, sadness, anger, jealousy, or guilt [2-6].

Previous research on adjustment of siblings of children with cancer found most siblings' general adjustment to be within normal limits [1]. However, a significant subset of siblings suffers from cancer-related posttraumatic stress symptoms (PTSS) [1,7,8], and there is evidence of poorer health-related quality of life (HRQoL) in this population [9-11]. School and social functioning may be impaired in the first time period after diagnosis [12-14]. In sum, the findings on psychosocial adjustment of siblings of pediatric cancer patients indicate that siblings do not suffer from severe psychopathology but are at risk for emotional, behavioral, and social problems, typically soon after the diagnosis [1,15].

Given the difficult circumstances that childhood cancer causes for all family members, it is important to understand the consequences of the diagnosis for siblings and to develop appropriate interventions to reduce distress and promote psychological adjustment. It is important to identify the needs of siblings and to recommend and encourage appropriate treatments when problems are detected [16]. However, intervention research with siblings is still rare [17]. The majority of published papers reported results of descriptive and correlational research, and there is a lot of non-empirical, anecdotal data. A previous review of empirically evaluated intervention programs showed improvements in siblings' depression symptomatology, medical knowledge about cancer, HRQoL, and high satisfaction ratings in siblings and parents [18]. But many existing studies are methodologically weak; most intervention studies relied on simple pre/post evaluations and only a minority used randomized controlled designs (RCTs) [18]. Concerning intervention timing, most studies used a broad inclusion criterion for the length of time since onset of cancer. Only one intervention so far reported targeting siblings of patients in an early stage of treatment and therefore aimed at prevention [19]. To our knowledge all published papers on empirically evaluated interventions with siblings used interventions in a group or camp format, and individual interventions were hardly ever evaluated in the literature.

The present study aimed at assessing the effect of a two-session early psychological intervention for siblings of pediatric cancer patients using a randomized controlled pilot trial. This intervention was provided early, i.e., within the first two months of diagnosis, and was conducted in an individual format. We expected that this intervention would be effective in the sense of yielding evidence that siblings who receive the intervention suffer from fewer anxiety and PTSS, report better HRQoL, and receive more social support compared to a control group with standard care.

## Methods

### Participants

Participants were recruited from June 2006 until October 2010 at two children's hospitals in Switzerland. Siblings had to meet all of the following criteria: (1) brother or sister with newly diagnosed childhood cancer, (2) medical treatment (inpatient or outpatient) necessary, (3) age of sibling from 6 to 17 years, (4) fluency in German. Families with a child who met criteria for inclusion were contacted within 1 month of diagnosis. If the family had several siblings who met the inclusion criteria, all willing siblings were included.

**Figure 1:**
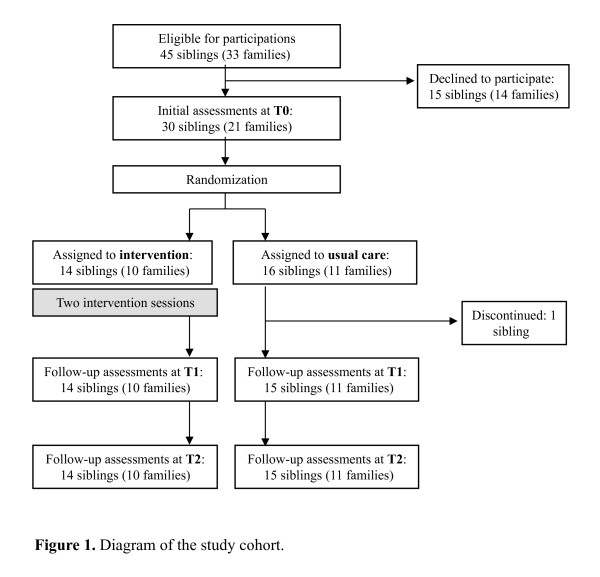
**Diagram of the study cohort**.

Forty-five siblings met the inclusion criteria and were asked to participate. Fifteen siblings and their families declined participation (33.3%); reasons were because siblings refused to participate (46.7%), because the study seemed too time-consuming (26.7%), or because parents thought the study would be an additional strain on the family (26.7%). Due to incomplete data at follow-up assessments (one drop-out), the final study sample comprised 29 siblings (response rate 64.4%) from 21 families (see Figure 1). The maximum number of siblings from the same family was three in each intervention arm. None of the ill children died during follow-up and none of the siblings had serious health problems during the study. Comparison of participants and non-participants revealed no significant differences in mean age of siblings (t = 0.47, p = .64), sex (χ^2 ^= 0.72, p = .40), type of diagnosis (χ^2 ^= 0.88, p = .64), intensity of treatment (Z = -0.31, p = .76), medical complications (Z = -0.22, p = .83), and health-related restrictions (Z = -0.50, p = .62).

A total sample size of 32 cases (16 for each group) would be required to detect a statistically significant effect size of 0.9 in a single tail t-test with a power of 0.80 and Type I error rate set to .05 [20]. Expected effect size was based on previous studies on sibling interventions [18] and on the notion that an individual intervention would achieve slightly higher effects.

### Procedure

The institutional review boards of both study sites approved the study. Written informed consent was obtained from parents and siblings older than 12 years. Assessments with all siblings were carried out at 4 to 6 weeks (T0; M = 36.0 days, SD = 10.2 days), 4 months (T1; M = 132.9 days, SD = 16.9 days), and 7 months (T2; M = 220.9 days, SD = 16.6 days) after the cancer diagnosis. The siblings were assessed by means of standardized individual interviews comprising the measures mentioned below. The interviews were conducted by trained psychologists. Questions were read aloud by the interviewer and corresponding answer scales were presented to siblings. The interviews lasted approximately 45 minutes, and most of them were conducted in the siblings' home; some were conducted at the hospital (8 out of 88 interviews in total). Mothers and fathers were independently assessed at the same time using questionnaires. Medical variables were retrieved from the responsible physicians. In return for participation, families received 50 Swiss francs (approx. 41 EUR/54 USD) after completing all three assessments.

The randomization list, stratified for sex, was generated by the program Rancode 3.6 (IDV, Gauting, Germany). The study used a cluster randomization scheme. The 21 participating families were randomly assigned to the intervention or the control group. Although multiple siblings per family could participate, randomization per family occurred just once and all the siblings received the same intervention. The randomization was stratified according to sex of the sibling closest in age to the child with cancer. Standard psychosocial care with the psycho-oncologist on the ward was available for both study groups. The utilization of psychosocial care by the end of data assessment was equally distributed between control and intervention group (U = 79.5; p = .82).

If the sibling was assigned to the intervention group, the first session of the intervention was conducted immediately after the T0 baseline assessment. The second intervention session was held 2 weeks after the first intervention session. A different interviewer, who was blind to the sibling's status in the project, conducted follow-up assessments at T1 and T2 with both control and intervention group.

### Measures

#### Medical knowledge

Medical knowledge about cancer was assessed using a scale that we developed. Siblings answered four questions: on name of the illness, medical understanding of the illness process, treatment options, and length of treatment. Using a comprehensive coding sheet, two clinical psychologists coded the answers together on a scale ranging from 0 to 2, with higher scores indicating better medical knowledge. Cronbach's alphas for the medical knowledge scores were satisfactory to poor, with α = 0.63 at T0, α = 0.50 at T1 and α = 0.34 at T2. In the current sample there was no correlation between age and medical knowledge at all time points and in both groups.

#### Social support

The number of individuals providing social support was assessed using a scale that we developed. Siblings received a comprehensive list of individuals that were available to them and might be sources of social support, including mother, father, grandparents, siblings, relatives, godmother and godfather, neighbors, close friends, peers, and teachers. They then indicated whether or not these individuals provided social support. The score was the total number of people providing support.

#### Health-related quality of life

HRQoL was assessed using KIDSCREEN-27, a standardized questionnaire for children from 8 to 18 years of age [21]. The KIDSCREEN instruments were developed and validated in several European countries simultaneously. KIDSCREEN-27 contains 27 items building five subscales: physical well-being, psychological well-being, autonomy and parents, social support and peers, and school environment. A 5-point Likert response scale is used in all subscales. In this study, we used both the child version and the parent version, filled out by mothers. All scores are reported as T-values, based on Swiss community norms, with higher scores indicating higher HRQoL. KIDSCREEN-27 was found to be a reliable and valid measure of HRQoL in children and adolescents [22]. In this sample, KIDSCREEN-27 showed very good internal consistency (self-report: α = 0.85 at T0, α = 0.88 at T1 and α = 0.89 at T2; proxy report: α = 0.87 at T0, α = 0.89 at T1 and α = 0.90 at T2). With the five siblings who had not reached the age of eight years at the T0 assessment, special care was taken that the questions were fully comprehended. The interviewer made sure that siblings understood the wording and explained specific terms if necessary.

#### Posttraumatic stress symptoms

Siblings were interviewed about their illness-related posttraumatic stress symptoms using the UCLA PTSD Reaction Index (UCLA RI) [23]. We altered the wording slightly to ensure that siblings reported on their experience of their brother or sister's cancer. The items of the UCLA RI closely follow the DSM-IV symptoms of posttraumatic stress disorder (PTSD) and can provide diagnostic information. Siblings were asked about their reactions during the past month and ranked their responses on a 5-point Likert scale from 0 (none of the time) to 4 (most of the time). An overall score was calculated (range 0-68), with higher scores indicating greater symptom severity. As to internal consistency of the UCLA RI, several reports found Cronbach's alpha to fall in the range of 0.90 [23]. In this sample, Cronbach's alpha was 0.90 at T0, 0.88 at T1, and 0.91 at T2 for the overall score.

#### Anxiety

The Spence Children's Anxiety Scale (SCAS) [24] is a 44-item self-report questionnaire to assess the severity of anxiety symptoms broadly in line with the DSM-IV dimensions of anxiety. It assesses six domains of anxiety: generalized anxiety, panic and agoraphobia, social phobia, separation anxiety, obsessive-compulsive disorder, and physical injury fears. The addition of all scores results in a total score. In this study we used the German version and norms of the SCAS [25]. Siblings rated the frequency with which they currently experienced each symptom on 4-point scale, with never (0), sometimes (1), often (2), and always (3). Internal consistency of the SCAS total score was excellent, with a Cronbach's alpha of 0.96 at T0, 0.93 at T1, and 0.94 at T2.

#### Socio-economic status

Socio-economic status (SES) as assessed by mothers was calculated by means of a 6-point SES-score of both paternal occupation and maternal education. Three social classes were defined as follows: scores 2-5, lower class; scores 6-8, middle class; and scores 9-12, upper class. This measure was used in previous studies and was shown to be a reliable and valid indicator of SES in Switzerland [26].

#### Medical variables

The pediatric oncologist in charge was asked to rate the following three medical variables concerning the ill child on a 3-point scale: *Intensity of treatment *(1 = low: surgery only or 6 months' chemotherapy only or both, with favorable prognosis; 2 = medium: treatment longer than 6 months according to the treatment protocol, with intermediate prognosis; 3 = high: treatment according to high risk protocols, bone marrow transplantation, with unfavorable prognosis), *Medical complications *(0 = no complications and good response to therapy; 1 = moderate complications, e.g., hospitalization due to infection; 2 = severe complications, e.g., multiple hospitalizations due to infections, no response to treatment) and *Health-related restrictions *(0 = no restriction; 1 = moderate restrictions, e.g., distinct fatigue, pain; 2 = severe, e.g., intense pain, considerable restrictions in physical and cognitive performance). The intensity of treatment and the medical complications items were used successfully in previous studies on children with cancer [27].

### Intervention

Our intervention was provided within the first two months after the cancer diagnosis and therefore during a stage which has been shown to be the most vulnerable time for siblings' adjustment [1,14]. By targeting a specific time frame, the intervention can be tailored to needs and stressors associated with this particular initial time period after diagnosis. Because in clinical practice it is hardly feasible to organize a group of siblings who are in the same stage of dealing the illness of their brother or sister with cancer, early preventive interventions are better held in an individual setting for practical reasons. Further, an individual format, even if manualized, allows more flexibility to run the intervention developmentally appropriate, to address personal concerns and to provide individualized cancer-related information.

We developed the standardized psychological intervention based on clinical experience, theoretical considerations, the relevant literature, and a qualitative pilot study that had gathered more information about siblings' experiences in this time period [28]. In two sessions, each approximately 50 minutes long, a clinical psychologist (first author of this paper) guided siblings and their parents through a three-step program: (1) medical information, (2) coping with stressful situations, and (3) information for parents.

The medical information part focused on the siblings' understanding of body functioning, the illness mechanism and location, and the cancer treatment, in particular chemotherapy. To accomplish this goal, pictures and storybooks were presented as aids. It was particularly emphasized that nobody was to blame for the development of cancer, nobody had done anything wrong, and cancer is not contagious. By learning more about the disease, treatment schedule, and side effects, siblings should gain a feeling of control over the situation which might reduce feelings of anxiety [29,30] and enhance social competence [31].

The part on coping encouraged siblings to think of changes and particular stressful situations in their life since the cancer diagnosis. Siblings individually chose the three most significant stressors. These were looked at in detail, and helpful coping strategies were discussed. Results of the coping session were written down on a special list and handed out to siblings. At least one parent joined after this part of the intervention. With the sibling's agreement, parents were informed about the relevant topics of the intervention in order to get as much support from parents as possible. Cognitive behavior therapy was the therapeutic approach used during problem identification and discussion of coping strategies. Following the coping and stress model proposed by Lazarus and Folkman [32] siblings were encouraged to appraise stressors in their daily life and develop coping strategies in response to their specific situation.

In the last part of the intervention, parents received a psychoeducational booklet developed by the authors containing information on the psychosocial situation of siblings of cancer patients in general and providing recommendations to parents on how to support siblings. At the end of each session there was time for questions from siblings and parents.

### Control condition

Families in the control group received standard psychosocial care, which consisted of meetings with the psycho-oncologist on the ward, who was primarily responsible for the ill child and the parents but also met with siblings if necessary. After follow-up assessments were completed, the control group was offered individual sessions for siblings. Two siblings made use of this.

### Statistical analyses

For data analysis we used statistical package SPSS for Windows, release 16 (SPSS Inc., Chicago, IL). All analyses were performed with two-sided tests. A *p *value < = .05 was considered significant. Kolmogorov-Smirnov Goodness of Fit Tests showed normality for all outcome measures. To compare nominal and ordinal scales, χ2 analyses and, when cells were too small, Mann-Whitney U tests were used. Normally distributed continuous data were analyzed using independent t-tests. To determine the effectiveness of the intervention, two-factor repeated measures analysis of variance (ANOVA) were performed. In the statistical analysis, siblings' adjustment variables were compared with respect to group, time, and group × time interaction. Post-hoc analysis for significant time effects was corrected for multiple comparisons using Bonferroni adjustment. If significant mean differences were detected, effect sizes (d) were calculated following Cohen [33].

## Results

### Sample characteristics and baseline assessment

Table 1 shows sample characteristics. The intervention and the control groups did not differ significantly on any demographic variables or on illness characteristics such as the type of the ill child's diagnosis (hematological malignancies vs. brain or other solid tumor) or the length of hospitalization. Likewise, none of the medical variables showed group differences at any assessment time point: Intensity of treatment (T0: Z = -0.61, p = .54; T1: Z = -0.25, p = .80; T2: Z = -1.08, p = .28), medical complications (T0: Z = 0.00, p = 1.00; T1: Z = -1.29, p = .20; T2: Z = -0.75, p = .45), health-related restrictions (T0: Z = -0.94, p = .35; T1: Z = -0.48, p = .63; T2: Z = -0.72, p = .47). Similarly, there were no significant between-group differences on any baseline outcome measure at T0: medical knowledge: t = 1.32, p = .20; social support: t = 1.17, p = .25; KIDSCREEN self-report: t = 1.26, p = .22; KIDSCREEN mother-report: t = 0.05, p = .96; UCLA PTSD: t = 1.39, p = .18; SCAS: t = 1.12, p = .27.

**Table 1:** Demographic and medical characteristics of the sample (N = 30)

	Intervention (N = 14)	Control (N = 16)	p*
**Age at baseline (years)**			
Median (Range)	8.5 (6-14)	11.5 (6-17)	.13

**Sex**			
Boys (%)	9 (64.3%)	9 (56.2)	
Girls (%)	5 (35.7)	7 (43.8)	.65

**Socio-economic status**			
Lower (%)	0 (0)	0 (0)	
Middle (%)	10 (71.4)	9 (56.2)	
Upper (%)	4 (28.6)	7 (43.8)	
**Median (Range)**	6 (4-6)	6 (4-7)	.78°

**Birth order**			
Younger (%)	4 (28.6)	8 (53.3)	
Older (%)	10 (71.4)	7 (46.7)	.18

**Type of diagnosis in ill child**			
Hematological malignancies (%)	7 (43.8)	10 (71.4)	
Brain or other solid tumor (%)	9 (56.3)	4 (28.6)	.13

**Days hospitalized**			
T0, **Median (Range)**	22 (14-37)	26 (4-35)	.69
T1, **Median (Range)**	40 (20-76)	41 (13-57)	.53
T2, **Median (Range)**	68 (10-100)	62 (17-94)	.19

* U-Tests according to Mann-Whitney for dichotomous variables, Chi-square analysis for categorical variables; °U-Test according to Man-Whitney based on SES score

Note: one twin is not included in the birth order data

At baseline the total sample did not differ from community norms on mother-reported HRQoL (t = -1.31, p = .20) and anxiety (t = 0.58, p = .57). But self-reported HRQoL at baseline was significantly lower than in the Swiss norm population (KIDSCREEN self-report: t = -2.38, p = .02). Further, the initial assessment identified 7 out of 30 siblings (23.3%) with a full and 13 (43.3%) with a partial DSM-IV related PTSD.

### Intervention effects

Table 2 presents the results of the repeated measure ANOVA.

**Table 2:** Means, standard deviations and analysis of variance for repeated measures (ANOVA)

	Intervention (N = 14)	Control group (N = 16)	ANOVA F (p)		
	
	M (SD)	M (SD)	Time (T)	Group (G)	T × G
**Medical knowledge**			5.11 (0.01**)	2.61 (0.12)	0.27 (0.77)
T0, mean score	1.45 (0.56)	1.23 (0.37)			
T1, mean score	1.46 (0.48)	1.28 (0.35)			
T2, mean score	1.63 (0.36)	1.37 (0.27)			

**Social support**			0.24 (0.79)	4.58 (0.41*)	0.69 (0.51)
T0, number	10.43 (1.16)	9.75 (1.88)			
T1, number	10.93 (0.83)	9.69 (2.73)			
T2, number	10.86 (0.77)	9.25 (2.86)			

**HRQoL: KIDSCREEN child version total score**			13.88(0.00***)	1.83 (0.19)	0.60 (0.55)
T0, mean T-scores	48.87 (4.79)	46.01 (6.75)			
T1, mean T-scores	52.95 (5.01)	49.12 (6.68)			
T2, mean T-scores	53.64 (6.94)	52.09 (7.36)			

**KIDSCREEN child version subscale psychological well-being**			37.16 (0.00***)	5.29 (0.03*)	1.57 (0.22)
T0, T-scores	39.59 (4.37)	36.69 (4.28)			
T1, T-scores	55.76 (8.45)	47.06 (8.96)			
T2, T-scores	54.76 (10.77)	52.08 (9.81)			

**HRQoL: KIDSCREEN parent version total score**			4.14 (0.02)*	1.11 (0.30)	1.49 (0.24)
T0, mean T-scores	48.70 (4.97)	48.60 (6.27)			
T1, mean T-scores	49.99 (5.16)	53.52 (6.22)			
T2, mean T-scores	49.20 (4.05)	51.68 (7.81)			

**Posttraumatic stress symptoms: UCLA RI**			4.15 (0.02)*	0.91 (0.35)	0.86 (0.43)
T0, total score	22.23 (14.06)	15.57 (11.89)			
T1, total score	15.00 (9.79)	13.71 (11.36)			
T2, total score	15.00 (13.04)	11.64 (11.45)			

**Anxiety: SCAS total score**			5.35 (0.01)**	0.64 (0.43)	1.38 (0.26)
T0, total score	29.14 (25.52)	20.47 (17.15)			
T1, total score	18.43 (12.35)	17.93 (15.62)			
T2, total score	19.71 (12.86)	15.33 (15.63)			

*p < = .05; **p < = .01; ***p < = .001

#### Medical knowledge

Results for the medical knowledge scale showed a main time effect (p = .01) with significant increase of knowledge from T1 to T2 (Table 2). No group or group × time effect could be seen in the ANOVA. However, comparison of mean knowledge levels of intervention and control groups at T2 revealed significantly better knowledge in the intervention group (M_int _= 1.62, SD_int _= 0.36; M_control _= 1.37, SD_control _= 0.27; t = 2.20, p = .04). Effect sizes concerning the group differences at T2 were in the medium range (T2: d = .78). No significant mean differences were found between groups at T0 and T1.

#### Social support

A significant main effect for group (p = .04) was found for the number of persons providing support, with the intervention group having a higher number of persons available. Time and group × time showed no effect on the social support measure. Effect sizes related to group differences were in the medium range (T1: d = .61; T2: d = .77).

#### Health related quality of life, child report

The KIDSCREEN child report total score revealed a significant time effect (p = < .001), with improvement of HRQoL from T0 to T1, T1 to T2, and T0 to T2 in both groups. The intervention had no significant influence on the KIDSCREEN total score as reported by siblings. However, the KIDSCREEN self-report subscale "psychological well-being" showed a significant main effect of group (p = .03) as well as a significant time effect (p = < .001), indicating improvements over time in both groups, but with siblings in the intervention group demonstrating better psychological well-being as compared to the control group. Effect sizes related to group differences were large for T1 (d = .99) and rather small for T2 (d = .26). Main effects of the intervention or effects of intervention × time interactions could not be found in any other subscale of the KIDSCREEN. Two more KIDSCREEN subscales in the child version showed significant improvements over time: "Autonomy and parents" (F = 3.95; P = .03) and "school environment" (F = 8.45; p = .001).

#### Health related quality of life; parent report

For the parent version of the KIDSCREEN no significant effect was noted with respect to group and group × time interaction. But the analyses showed a significant time effect with significant improvement of HRQoL from T1 to T2 and T0 to T2.

#### Posttraumatic stress symptoms

ANOVA results showed a significant time effect (p = .02) in both groups on the UCLA RI scales. However, the time effect was no longer significant in the post-hoc pairwise comparisons using Bonferroni tests. There was no main effect for group in posttraumatic stress symptoms. Full PTSD diagnosis decreased in the whole sample, with 5 siblings (16.7%) that met full diagnosis criteria at T1 and 3 siblings (10%) at T2.

#### Anxiety

Siblings' anxiety showed a time effect (p = .02) with a significant reduction of anxiety in both groups from T1 to T2. No group or time × group effect could be found. A look at the baseline T0 scores of anxiety and PTSS shows that the intervention group starts out with a considerably higher level although not statistically significant. We therefore also conducted repeated measures ANCOVAS with T0 scores of anxiety and PTSS as covariates. But still no group effect could be shown.

## Discussion

The aim of this pilot trail was to evaluate the effectiveness of an early psychological intervention with siblings of newly diagnosed pediatric cancer patients. Although generalizability of our results is restricted due to the small sample size, this study provides preliminary evidence that a two-session sibling intervention has a positive impact on psychological well-being, social support and medical knowledge of siblings. However, no intervention effect could be found with regard to proxy-rated HRQoL, PTSS, and anxiety.

Since children often rely on their own interpretations of illness and sometimes have a distorted picture of it, it is crucial to provide age-appropriate medical information [34]. We found a better medical knowledge score in siblings in the intervention group half a year after the cancer diagnosis compared to the control group. Although one needs to be cautious not to overinterpret the findings of our pilot trial, this might indicate an effect of our intervention on medical knowledge. This beneficial finding is in line with previous intervention studies [30,35]. Although this study did not examine the effects of better medical knowledge, other researchers found that enhanced knowledge had a positive impact on siblings' adjustment and social competences [29,31].

The number of individuals available for social support was elevated for siblings who took part in the intervention. This improvement could possibly be related to the coping part of our intervention, where many of the coping skills discussed involved looking for social support. Moreover, the psychoeducational booklet provided to parents covered social support issues. Although having a higher number of available individuals does not necessarily mean better social support, but having a greater number of available potential partners increases chances of receiving helpful social support, especially for our population, which is confronted with decreased social resources in the core family due to the cancer diagnosis [1]. Other studies identified social support as an important construct that may play a critical protective role in the psychosocial adjustment of siblings of cancer patients [36,37]. Our study is the first to include social support as an outcome measure in the evaluation of interventions for siblings.

Siblings' self-reports indicated a better psychological well-being after the intervention. Even though this was the only subscale of the KIDSCREEN questionnaire showing group differences, well-being represents an essential part of siblings' psychological adjustment (positive emotions, satisfaction with life, and balanced emotionality). Positive effects on HRQoL were also found in two other studies [11,38]. Notably, in our study this result was not apparent from the mothers' reports of the child's HRQoL. This might be due to parents' difficulties in judging the siblings's HRQoL in an emotional domain such as psychological well-being [39].

Contrary to our hypothesis, anxiety and PTSS were not improved by the intervention, although trends in the desired directions could be observed. Anxiety was not clinically increased in our sample compared to norms, a finding also seen in previous studies [9,35,36], and might therefore be an inappropriate measure to assess effects of an intervention. PTSS, on the other hand, was high at baseline, with almost a quarter of our sample qualifying for full diagnosis of PTSD. These findings are in line with previous studies that did not operate with DSM-IV related instruments but revealed similar high numbers of PTSS scores [7,8,36,38]. However, our intervention might have been too unspecific regarding PTSS to help siblings with relevant symptoms.

Alternative explanations for absent intervention effects should also be considered. It is possible that families' involvement in the study and the data collection itself increased and perhaps improved communication between parents and siblings [40] and therefore led to better adjustment in both study groups. Other unspecific factors may also play an important role. By enrolling siblings in a specific sibling program, parents may demonstrate their concern for them and may develop particular efforts to spend time with them [41]. Likewise, we have to consider that our whole population had standard psychological care at hand, and this might have leveled group differences. It is also possible that our age range was too broad; whereas certain age groups could have benefited from the intervention, for others the intervention may not have been appropriate.

This study has a number of limitations. First, the fact that our intervention was for the early time period after diagnosis made recruitment more difficult and resulted in a rather small sample. The study has therefore limited power to detect group differences and we were not able to perform subgroup analyses (age, sex). Therefore, findings of this pilot study are preliminary and exploratory and should be interpreted with caution. Second, we developed the measures for social support and medical knowledge ourselves. Internal consistencies of these measures were rather poor and further psychometric criteria were not evaluated. Third, The KIDSCREEN-27 scale is not validated for children below eight years of age and the youngest study participants of our study were six years old. However, since the KIDSCREEN-27 questionnaire was included in an interview, the interviewer could pay special attention to language skills. Advantages of the KIDSCREEN-27 are the little number of items, the availability of a parent version, existence of a German version and Swiss norms. Finally, families with lower socio-economic status were not represented in the sample. This might be due to the inclusion criterion of fluency in German, which excluded families with an immigrant background.

Despite these limitations, this study has several strengths, including its randomized controlled prospective design and the manualized intervention for the early stage after diagnosis. Moreover, statistical conditions were good, with no socio-demographic differences between study participants and non-participants, no differences between intervention and control groups on all baseline scores, and almost no attrition.

## Conclusions

The early, two-session individual psychological intervention evaluated here is a promising approach for siblings in several respects. Efforts to provide medical information to siblings, to enhance their coping skills, and to inform parents about their situation seemed to be rewarded by increased medical knowledge, increased sibling-reported psychological well-being and more social support resources. No intervention effects could be found with regard to anxiety, PTSS, and parent-reported HRQoL. To overcome the problem of small sample size, future research should aim for multicentric studies. Also, based on the considerably high rates of PTSS, interventions should be more trauma-focused.

## Competing interests

The authors declare that they have no competing interests.

## Authors' contributions

AP was involved in the conception and design of the study, in data collection, conducted the interventions, performed the data analysis and drafted the manuscript. AG made contributions to design of the study and the acquisition of data. EB participated in data acquisition and revised the manuscript for pediatric content. MAL made substantial contributions to conception and design, supervised all aspects of the study and revised the final draft of the manuscript. All authors read and approved the final version of the report.
